# Practice (Doesn´t) Make Perfect Shooters: The Influence of Experience on Penalty Execution in Elite Soccer

**DOI:** 10.5114/jhk/205224

**Published:** 2025-11-19

**Authors:** Rubén Maneiro, Antonio Ardá, José Luís Losada, Iyán Iván-Baragaño

**Affiliations:** 1 Department of Special Didactics, University of Vigo, Vigo, Spain.; 2 Department of Physical and Sport Education, University of A Coruña, A Coruña, Spain.; 3 Department of Social Psychology and Quantitative Psychology, University of Barcelona, Barcelona, Spain.; 4 Research Group and Innovation in Designs (GRID), Technology and Multimedia and Digital Application to Observational Designs, Autonomous University of Barcelona, Barcelona, Spain.; 5 Department of Sport Sciences, Faculty of Medicine, Health, and Sports, Universidad Europea de Madrid, Madrid, Spain.

**Keywords:** set piece, performance analysis, men´s soccer, observational methodology

## Abstract

The aim of this study was twofold: firstly, to identify the variables associated with the experience of penalty shooters, and secondly, to understand the interaction of contextual variables and penalty execution that differentiated experienced from inexperienced players. To achieve this, a systematic observational methodology was employed to analyze 1,589 penalty kicks in various international club and national team competitions for men. The analyses were conducted using contingency tables and the chi-square statistic, as well as decision trees and binary logistic regression, to address the second objective. Categorizing players as experienced or inexperienced was based on their role as penalty kick takers in the team. Considering experience, statistically significant differences were found in contextual variables such as the specific position, age, timing, and match status. Similarly, differences were found in the shooter's running approach and kick height. At a multivariate level, both classification models showed significant differences in shooting patterns based on experience: players differed in terms of match timing, the specific position, the running approach, and the penalty outcome. The results of this study demonstrated differences between experienced and inexperienced players, highlighting the need to: i) delve deeper into research on this specific soccer action, and ii) enhance training of such actions among a greater number of players, not solely relying on experienced players for their execution.

## Introduction

One of the most renowned phrases in the world of high-level soccer is the statement “penalties are a lottery”. Many of the errors or successes of the shooter were attributed to chance or factors beyond the control of the variables involved in a penalty kick. In recent years, and with the impetus provided by research focused on this type of action, scientific evidence has demonstrated that success in penalties can be attributed to specific variables that can be measured in the laboratory and executed during competition (Dicks et al., 2010a; Piras and Vickers, 2011; Wood et al., 2015).

The penalty kick is one of the set-piece actions in soccer that occurs less frequently (one every two matches, on average) but with greater effectiveness (between 70 and 85% are scored), according to scientific literature (Carling et al., 2005; Noël et al., 2021). Furthermore, more than 20% of knockout-based matches are decided thanks to this type of action (Jordet et al., 2007). The penalty kick is based on the mutual personal interaction between two actors (the shooter and the goalkeeper), with diametrically opposed intentions, where the behavior of each influences the opponent's conduct (Navia and Ruiz, 2014). It is an (imbalanced) duel of intentions, where the success of one leads to the failure of the opponent (Lopes et al., 2012). Scientific literature focused on this interaction reports that the ball takes between 408 and 620 ms to reach the goal (Sánchez et al., 2005), and the goalkeeper needs between 700 and 1000 ms to reach the ball, highlighting the goalkeeper's temporal disadvantage in this type of action (Dicks et al., 2010b).

In this regard, studies have been conducted on different technical and biomechanical aspects in penalty kick execution. Thanks to these studies, it is possible to know that a reduced speed when approaching the ball for striking results in greater accuracy (Lees and Nolan, 2002), that striking with the inside of the foot is more precise (Hunter et al., 2018), and that left-footed players tend to aim for the left sector, while right-footed players tend to aim for their right (Barbero et al., 2023).

On the other hand, one of the significant decisions that coaches must make is which player takes the penalty kick. Although scientific literature focused on the "specialist player" is scarce, the work of Jordet et al. (2007) concludes that forwards are more likely to score goals than midfielders or defenders, due to the emotional relationship forwards have with scoring and their greater ability to cope with pressure situations (Navia and Ruiz, 2014; Wilson et al., 2009). Another important decision is the order of players during penalty shootouts in knockout competitions. One of the significant discoveries in recent years has been which team should take the first penalty kick. In this regard, the work of Apesteguia and Palacios-Huerta (2010) observed that the team shooting first had a 60% chance of winning the shootout, a probability that increased to 66% when the shootout was decided in the 5 standard rounds. Regarding the order of shooters, the work of McGarry and Franks (2000) suggests arranging shooters by their quality in reverse order, meaning placing "expert" players in reverse order to the shootout (the best shooter in the 5^th^ position, the second best in the 4^th^, and so on). The work of Rudi et al. (2020) proposes the inclusion of the classic ABAB order into ABBA order to reduce the influence of the first shooter.

In light of the results, the importance of weekly training for these actions should be a reality during competitions. One of the major decisions teams must make is which player should assume the responsibility of taking the penalty kick. Coaches are responsible for selecting the most talented players or those with greater technical skills for this task. Therefore, the objectives of the present study were, on the one hand, to identify the variables that would characterize and differentiate the experienced player from the inexperienced one; and, on the other hand, to understand the interaction of the variables associated with the context and execution of penalty kicks that would help differentiate experienced from inexperienced shooters.

## Methods

### Participants

A total of 1589 penalties executed during different national and international championships were analyzed, such as the last three World Cups, the last three UEFA Euros, the last five editions of the Champions League, and the main European soccer leagues. Matches were recorded from public images broadcasted on television, and through a post-event record, thus ensuring respect for behavior spontaneity, as well as the registration in its natural environment. According to the Belmont Report (1979), the use of public images for research purposes does not require consent.

The term “habitual or experienced shooter” is defined as the first or the second habitual shooter of the teams, with “non-habitual shooters” being the other players in the squad.

### Measures

An observation instrument constructed *ad hoc* for the present research was designed. The observation instrument (Table 1) consisted of 15 criteria and 42 categories.

**Table 1:** Observation instrument.

CRITERIA	CATEGORIES	DESCRIPTION
Position	Striker	The shooter is a forward.
	Midfielder	The shooter is a midfielder.
	Defense	The shooter is a defender.
	Goalkeeper	The shooter is a goalkeeper.
Experience	Yes	The shooter is the regular penalty taker.
	No	The shooter is not the regular penalty taker.
Age	>28	The shooter is over 29 years old.
	23–28	The shooter is between 23 and 28 years old.
	<23	The shooter is under 23 years old.
Home	Home	The penalty is taken by the home team.
	Neutral	The penalty is taken on a neutral field.
	Away	The penalty is taken by the away team.
Time	0–30	The penalty is taken in the first 30 minutes of the match.
	31–60	The penalty is taken between the 31^st^ and the 60^th^ min of the match.
	61–90	The penalty is taken between the 61^st^ and the 90^th^ min of the match.
	Extra Time	The penalty is taken in extra time.
	Penalty Shootout	The penalty is part of a penalty shootout.
Match	Match	The penalty is taken during the match.
	Penalty Shootout	The penalty is part of a penalty shootout.
Side	Right	The penalty is taken to the right.
	Center	The penalty is taken to the center.
	Left	The penalty is taken to the left.
Height	Low	The penalty is taken below the goalkeeper's waist.
	Medium	The penalty is taken between the goalkeeper's waist and head.
	High	The penalty is taken above the goalkeeper's head.
Footednes	Right footed	The penalty is taken with the right foot.
	Left footed	The penalty is taken with the left foot.
Race length	Short	The shooter's run begins inside the penalty area.
	Long	The shooter's run begins from outside the penalty area.
Race type	Continuous	The shooter's run is continuous.
	Discontinuous	The shooter's run includes interruptions.
	Low	The goalkeeper attempts to save with a low dive.
Match Status	Winning	The shooter's team is winning.
	Drawing	The shooter's team is drawing.
	Losing	The shooter's team is losing.
Penalty outcome	Goal	The penalty results in a goal.
	No goal	The penalty does not result in a goal.

### Design and Procedures

To achieve the proposed objectives, the observational methodology (Anguera, 1979) was utilized. Among the possible designs that observational methodology can present, a nomothetic (several units of study corresponding to the analyzed players), intersessional follow-up (across different competitions), and multidimensional (multiple levels of response) design was applied (Anguera et al., 2017; Anguera and Hernández-Mendo, 2016). The systematic observation carried out was non-participant and active, utilizing an observational sampling “all occurrence” (Losada and Manolov, 2015). The study of physical and sports activity offers vast possibilities for research planning, considering observation as a mixed method in itself (Anguera, 2020)

The observational methodology has a good fit in the analysis of behaviors in soccer, mainly due to the following characteristics (Anguera et al., 2017, 2018, 2020): the degree of intervention and manipulation is zero, the study of behavior is based on terms of spontaneity and genuineness, the behavior occurs in its natural context, guaranteeing the absence of intrusively caused alterations. This methodology is integrated within the mixed methods perspective (Bazeley and Kemp, 2012; Creswell and Plano-Clark, 2007; Johnson et al., 2007) since it allows the integration of qualitative and quantitative data.

Prior to the coding process, and to reduce interobserver variability, eight training sessions were carried out, following Anguera et al. (2018) and Manolov and Losada (2017). In the first place, four observers were selected for data collection, three of them were PhDs in Sports Sciences, who were also UEFA PRO soccer coaches, and with experience in studies of this type. To ensure methodological quality, one of the co-authors, an expert in observational methodology, ensured that all the methodological steps were correct. Secondly, the training sessions lasted one hour each. The first three sessions were carried out in groups with the selected observers. The theoretical approach of this study was presented to them, the behaviors to be observed were delimited, the observation instrument was exposed and the observers were trained in the use of the Lince Plus recording instrument (Soto et al., 2019). The fourth session consisted of the observation and recording by the observers of 10 penalties previously selected by the principal investigator, ordered from the least to the greatest complexity. Once the actions were recorded by those observers, the discrepancies found were discussed. The fifth and sixth sessions were carried out individually with each of the observers. The delimitation of the recorded actions was carried out previously by the principal investigator and those observers who were instructed in the recording of the actions. The last two sessions were also carried out individually and during them, the concordance coefficient of Cohen's Kappa (1960) was verified between the principal investigator and each of the observers. Finally, two files were given to each of the observers with the offensive actions under analysis. The actions used to obtain the value of the coefficient of agreement represented 10% of the total actions (n = 154). The analysis of these actions was carried out individually and sent to the principal investigator of the study.

Data quality control was carried out using the IBM SPSS Statistics 25 program by means of an interobserver concordance analysis by the Cohen’s Kappa coefficient for each of the criteria, the overall value being excellent (0.91) according to the scales of Fleiss et al. (2013) (Table 2).

**Table 2:** Data quality control. Cohen's kappa values.

Criterion	O1O2	O1O3	O1O4	O2O3	O2O4	O3O4	Kappa
Position	1	1	0.94	1	0.913	0.879	0.958
Experience	1	1	1	1	1	1	1
Age	1	1	1	1	1	1	1
Home	1	0.981	0.971	1	1	0.888	0.972
Time	1	0.953	1	1	1	1	0.992
Side	0.875	0.842	0.877	0.864	0.848	0.823	0.854
Height	0.812	0.834	0.911	0.821	0.795	0.723	0.854
Footedness	1	1	1	1	1	1	1
Race length	1	1	1	1	1	0.917	0.986
Race type	0.928	0.934	0.961	0.832	0.841	0.939	0,905
Match Status	0.846	0.855	0.862	0.972	0.923	0.993	0.908
Kappa	0.928	0.928	0.928	0.857	0.914	0.915	0.91

### Statistical Analysis

First, a bivariate analysis of association was conducted between the criterion "experience" and the rest of the criteria analyzed. The existence of statistical dependence was tested using the chi-square test and the contingency table procedure with a significance level of *p* < 0.05. When a statistically significant association was observed, the effect size was calculated using the contingency coefficient, categorized as small (ES = 0.10), medium (ES = 0.30), or large (ES = 0.50).

In relation to the second objective, a binary logistic regression and a binary decision tree classification model were performed. Prior to model fitting, hyperparameter tuning was performed to maximize model performance, measured from the classification table, and to avoid overfitting and underfitting on the validation sample. The final model was trained on 60% of the total sample, with validation on the remaining 40% of cases, introducing the variable “experience” as dependent and the others as predictors. The growth method was CHAID (Chi Square Automatic Interaction Detection). The maximum depth of the model was set at 4 levels, and the minimum number of observations in the nodes was 100 and 50.

**Figure 1:**
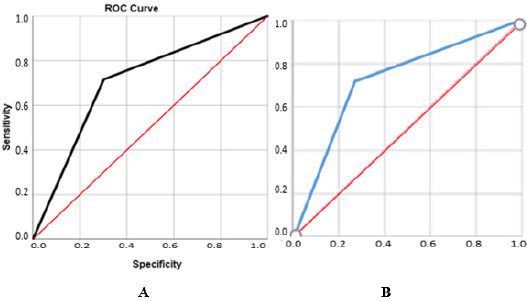
ROC curve for the decision tree (A) and binary logistic regression (B).

Finally, the model performance was evaluated based on the area under the ROC curve (AUC), presented in Figure 1 and considered excellent (0.90 < AUC < 1.00), good (0.80 < AUC < 0.90), fair (0.70 < AUC < 0.8), poor (0.6 < AUC < 0.7), or fail (0.5 < AUC < 0.6) (Marzban, 2004).

## Results

Table 3 presents the bivariate results between the experienced or habitual player (yes/no) and different variables considered. As can be seen, there were eight variables that showed a statistically significant relationship: “*Position*” (χ^2^ = 172.88; *p* < 0.001), “*Age*” (χ^2^ = 45.48; *p* < 0.001), “*Home*” (χ^2^ = 31.47; *p* < 0.001), “*Time*” (χ^2^ = 154.25; *p* < 0.001), “*Match*” (χ^2^ = 152.94; *p* < 0.001), “*Height*” (χ^2^ = 11.70; *p* = 0.003), “*Race Type*” (χ^2^ = 9.30; *p* = 0.002), and “*Match Status*” (χ^2^ = 47.40; *p* < 0.001).

**Table 3:** Relationship between the experienced player (yes/no) and different variables of interest.

CRITERIA		Experience				
		% Yes	% No	χ^2^	Sig.	Cont. Coef
Position	Goalkeeper	3.1 (0.0%)	1.9 (100%)	172.88	<0.001	0.33
	Defender	81.9 (21.1%)	51.1 (78.9%)			
	Midfielder	382.4 (54.1%)	238.6 (45.9%)			
	Forward	479.7 (74.8%)	299.3 (25.2%)			
Age	>28	68.3 (35.1%)	42.7 (64.9%)	45.48	<0.001	0.17
	23–28	527.1 (60.3%)	328.9 (39.7%)			
	<23	351.6 (68.7%)	219.4 (31.3%)			
Home	Local	527.7 (63.9%)	329.3 (36.1%)	31.47	<0.001	0.14
	Neutral	43.1 (87.1%)	26.9 (12.9%)			
	Visiting Team	376.2 (55.3%)	234.8 (44.7%)			
Time	0–30	139.2 (71.7%)	86.8 (28.3%)	154.25	<0.001	0.317
	31–60	248.1 (71.7%)	154.9 (28.3%)			
	61–90	308.5 (69.5%)	192.5 (30.5%)			
	Extra time	1.2 (100%)	0.8 (0.0%)			
	penalty shootout	250.0 (36%)	156.0 (64%)			
Match	Match	697.0 (70.8%)	435 (29.2%)	152.94	<0.001	0.31
	penalty shootout	250.0 (36.0%)	156 (64%)			
Side	Right	365.5 (61.5%)	222.5 (38.5%)	0.008	0.996	--
	Down the middle	148.4 (61.8%)	92.6 (38.2%)			
	Left	442.1 (61.6%)	275.9 (38.4%)			
Hight	penalty shot high	200.1 (53.5%)	124.9 (46.5%)	11.70	0.003	0.08
	medium height	253.7 (65%)	158.3 (35.0%)			
	penalty shot low	493.2 (63%)	307.8 (37.0%)			
Footednes	left-footed	220.4 (60.3%)	137.6 (39.7%)	0.30	0.58	--
	right-footed	726.6 (61.9%)	453.4 (38.1!)			
Race length	short race	644.1 (60.3%)	401.9 (39.7%)	2.15	0.14	--
	long race	302.9 (64.2%)	189.1 (358%)			
Race type	continuous race	790.6 (59.9%)	493.4 (40.1%)	9.30	0.002	0.07
	stop-and-go race	156.4 (70.1%)	97.6 (29.9%)			
	dark colors	359.0 (62.3%)	224.0 (37.7%)			
Match status	winning	242.0 (70%)	151.0 (30%)	47.40	<0.001	0.17
	drawing	502.4 (53.6%)	313.6 (46.4%)			
	losing	202.6 (71.4%)	126.4 (28.6%)			
Penalty outcome	Goal	727.8 (62.7%)	454.2 (37.3%)	2.69	0.10	--
	No goal	219.2 (57.9%)	136.8 (42.1%)			

### Decision Tree Results

The CHAID classification tree showed a total of 14 nodes, out of which 8 were terminal. The tree had 4 levels (level 1: match; level 2: position; level 3: age and race length; level 4: move outcome). The model correctly classified 68.3% of the overall cases. Specifically, for each dependent category of the criterion, it provided a higher success rate for the "yes experienced" category with 88.7%. The area under the curve (AUC) of the model was 0.707 [95% CI = 0.674–0.740].

**Figure 2:**
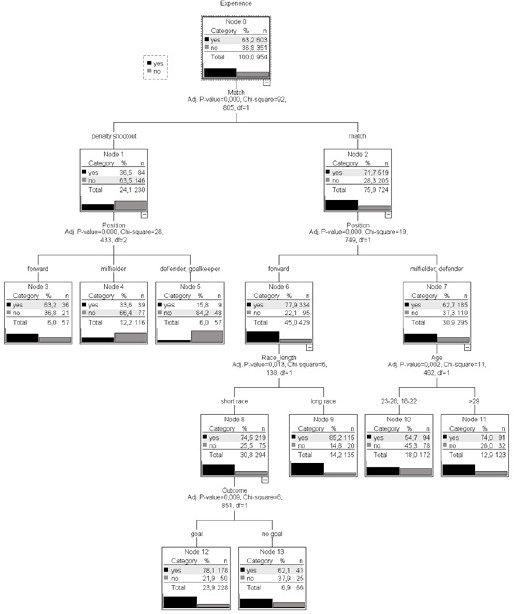
Decision tree.

The optimized decision tree (Figure 2) was presented using the "test" sample. The first node corresponded to the criterion “experience of the shooter”, which was the dependent criterion. In this node, a higher percentage of penalty kicks was observed to be executed by experienced players (first or second habitual shooter), with 63.2% for experienced players and 36.8% for inexperienced players. This criterion branched into 2 nodes, node 1 and node 2, belonging to the criterion “match” or “penalty shootout”, indicating that this criterion was the main predictor (χ^2^ = 92.805; *p* < 0.001). Node 1 comprised the categories “yes” and “no” of the criterion “penalty shootout”, with a higher occurrence in “no” at 63.5% of cases, compared to node 2, which consisted of the criterion “match” and the categories “yes” and “no”. Here, there was a higher occurrence in “yes” for 519 observations, totalling 71.7%. The next criterion introduced by the algorithm from node 1 was “position” (position or role of the penalty shooter) (χ^2^ = 28.433; *p* < 0.001), which branched into nodes 3, 4, and 5 (all terminal). These nodes showed that in penalty shootouts, the experienced player taking the penalty was a “forward” with a percentage of 63.2%, significantly decreasing for players in the “midfielder” position (33.6%) and “defender/goalkeeper” (15.8%).

In node 2, the next criterion presenting the highest information gain was again “position” (χ^2^ = 19.749; *p* < 0.001), showing that the highest probability of penalty kicks by experienced players during matches was 77.9% for “forward” (node 6), decreasing to 62.7% for “midfielder” and “defender” (node 7). From node 6, nodes 8 and 9 branched out, corresponding to the criterion “race length” (χ^2^ = 6.138; *p* = 0.013). In this case, there was a higher probability of “long race” (85.2%) in node 9 when the penalty kick was taken by the experienced forward during matches, compared to “short race” (74.5%) (node 8). While node 9 was terminal, from node 8, the criterion “outcome” (χ^2^ = 6.851; *p* = 0.009) branched out, including nodes (categories) 12 and 13, both terminal. Specifically, there was a higher probability of “goal” (78.1%) when the race was short and executed by the forward during matches. Finally, nodes 10 and 11 branched out from node 7, which referred to the age of the penalty shooter (χ^2^ = 11.462; *p* = 0.002). Specifically, 74% of successful penalties executed during matches were taken by “midfielders” or “defenders” who were over 29 years old, a percentage that decreased to 54.7% when they were executed by younger players (18–28 years old). These last two nodes were terminal.

### Binary Logistic Regression Results

A total of 8 variables were included in two preliminary models using backward and forward Wald Statistic, all of which were statistically significant in the bivariate Chi-square analysis (Table 4). For the final model, four variables showed the greatest increase in the odds ratio favouring the player's experience. The independent variables “position midfielder” and “position forward” increased the odds ratio for the “experienced” category by 8.048 times and 2.231 times, respectively, compared to the reference category. Additionally, the independent variables “age 23–28” and “age > 29” increased the odds ratio for the “experienced” category by 3.925 and 1.652 times, respectively.

**Table 4:** Results of the binary logistic regression model.

	B	Error ES	Wald	df	*p*	Exp (B)
Position			83.811	3	0.000	
Position (1)	21.19	17537.285	0.000	1	0.999	1596977352.759
Position (2)	2.085	0.260	64.229	1	0.000	8.048
Position (3)	0.803	0.124	42.138	1	0.000	2.231
Age			36.906	2	0.000	
Age (1)	1.367	0.240	32.508	1	0.000	3.925
Age (2)	0.502	0.128	15.390	1	0.000	1.652
Home			37.189	2	0.000	
Home (1)	−0.287	0.121	5.670	1	0.017	0.750
Home (2)	−2.360	0.398	35.126	1	0.000	0.094
Match (1)	−1.148	0.141	66.036	1	0.000	0.317
Constante	−0.311	0.181	2.954	1	0.086	0.733

Conversely, the independent variables that decreased the odds ratio were “home” and “match”. Specifically, “neutral field” and “visiting field” decreased the odds ratio by 0.750 and 0.094 times, respectively compared to the default category (local field). Finally, the variable “match penalty shootout” reduced the odds ratio by 0.317 times.

The model had specificity of 46.4% and sensitivity of 89%. The overall classification accuracy was 72.6%. The model was well-fitted based on the result of the Nagelkerke's R^2^ = 0.264.

## Discussion

The objectives of this study were, on the one hand, to identify the variables that differentiated the experienced player from the inexperienced one, and on the other hand, to understand the interaction of variables associated with the context and execution of penalties that would help distinguish between experienced and inexperienced shooters. The overall prediction accuracy of the model was adequate. The model yielded good evaluation metrics and allowed the study to focus on the explainability method.

The available results allowed to ascertain that at a bivariate level, there were statistically significant associations that differentiated the experienced or habitual shooter from the other non-habitual shooters. Specifically, it is possible to affirm that habitual players were usually forwards (74.8%), and furthermore, they had more years of experience, as almost 69% were aged 29 years or older. This corroborates the work of Wilson et al. (2009) on the greater ease of forwards in handling pressure situations, emotional responsibility in stressful situations, and their direct relationship with scoring goals.

Regarding the timing of the match, significant differences were observed between whether the penalty was taken during a regular match or during a penalty shootout. Specifically, it can be affirmed that while during matches the majority of shooters were experienced (with a percentage of above 70%), during penalty shootouts, and due to the particular nature of these kicks, this percentage decreased drastically to 36%. The protocol in these situations indicates that five kicks should be taken, which gives the opportunity for inexperienced players to take different kicks. In this regard, as McGarry and Franks (2000) pointed out, expert shooters should take the last two kicks, as they are more crucial and decisive for achieving success, being more familiar with the pressure and stress associated with these moments.

On the other hand, although no statistical differences were found regarding the side of the kick (right, center or left), significant differences were found in the height of the kick. Despite previous studies recommending that successful kicks should target the upper third of the goal (Bar-Eli and Azar, 2009; Horn et al., 2021), our findings do not reveal a fixed pattern. Instead, experienced shooters adopted a mixed strategy, aiming almost equally at different heights of the goal. This pattern of unpredictable behavior aligns with predictions from game theory, adhering to the Nash equilibrium of mixed strategies (Azar and Bar-Eli, 2011).

One of the variables scarcely studied in penalty kicks is the type of the approach of the shooter, whether it is a continuous or a discontinuous approach in their movement towards the ball to execute the kick. In the present study, solid evidence was found indicating that experienced players performed a “stop and go” approach in 70% of cases, compared to only 30% of such cases in inexperienced players. One possible explanation from a biomechanical perspective could be that this type of approach allows constant adjustment of their steps to improve ball striking. Another possible explanation from a perceptual perspective is that it allows them to visualize the goalkeeper's movements, thus obtaining information about their behavior. That is, the experienced shooter manages their kick based on the goalkeeper's movements. Meanwhile, the inexperienced shooter makes decisions moments before the kick, ignoring potential information that may come from the goalkeeper. This variable still requires further research to find causal relationships between the type of movement and the final success.

Regarding the partial match result at the time of the penalty kick, greater percentages of habitual shooters were observed in two important moments of the match. When the team was winning or losing, over 70% of the kicks were taken by experienced players, a percentage that decreased to 53% when the match was tied. Thus, it is possible to think that experienced players assume responsibility for penalty execution in decisive moments of the match, either to consolidate the lead or to achieve an equalizer.

At the multivariate level, the results of the decision tree consolidate the findings from the bivariate analysis and allow differentiation of the particular shooting strategies by experienced and inexperienced players during matches. On the one hand, during penalty shootouts, inexperienced players took 63% of the kicks, compared to only 36% taken by experienced players. This can be interpreted in two complementary ways: first, teams were facing a decisive situation during the competition (penalty shootouts), where success or failure was decided, with players who did not have sufficient technical, psychological, and emotional capabilities to handle these situations. As depicted in Figure 2, teams did not have experienced shooters in positions such as midfielders or defenders. This scenario was already highlighted by McGarry and Franks (2000) some years ago, where they warned about the need for careful selection of shooters and the importance of training for all team players. Navia and Ruiz (2014) recommended recreating these conditions as much as possible during training, introducing elements such as initial draw, player order selection, following usual mechanics, and adding some form of psychological pressure in the form of rewards or punishments. It should also be noted that the pressure experienced in a penalty shootout is difficult to replicate in training sessions. Second, during regular matches, the results were more encouraging. While forwards monopolized the kicks (78%) as habitual players, there was an increase of over 60% in experienced midfielders and defenders. However, two complementary peculiarities were observed: while the former scored goals in 80% of the occasions when they made short approach runs to the ball (which might imply more accurate kicks, according to Lopes et al., (2012)), the latter were over 29 years old when they became “habitual shooters”. That is, during matches, when penalties were not taken by forwards, coaches preferred older players, with more years of experience and more sporting background to face these situations. The variable of age in this type of kicks has been addressed in another retrospective study (Almeida et al., 2016), with inconclusive results as of yet.

Finally, the results of the binary logistic regression confirmed the previous results and reinforced in terms of odds that the most important variables in relation to the shooter's experience (Yes/No) are their field position (midfielders and forwards), as well as the shooter's age.

## Conclusions

In high-performance soccer, it is necessary to master all facets of the game. Penalty kicks are situations that, although they occur infrequently during matches, have a very significant impact on the final outcome of games and competitions. The main conclusions derived from this study can be summarized in three points: first, there are still very important differences in the variables associated with the experienced player compared to the inexperienced one; second, players are more prepared to face a penalty kick during matches than during the penalty shootout phase; finally, teams should include training programs for all squad members, with special attention to midfielders and defenders. By implementing these considerations, the potential for success in these actions can be increased, and consequently, during different competitions.

## Future Research Directions

The main future research directions emerging from this manuscript could be summarized as follows: (1) including more diverse participants, such as youth teams or women's soccer would be recommended; (2) including psychological and environmental factors or variables associated with the goalkeeper behaviour could provide new insights; (3) considering longitudinal designs could provide more information on how experience and other variables influence penalty taking over time.
